# Operationalising reproducibility in soft robotics

**DOI:** 10.3389/frobt.2026.1751222

**Published:** 2026-02-10

**Authors:** David Howard

**Affiliations:** CSIRO Robotics, Brisbane, QLD, Australia

**Keywords:** benchmarking, embodied robotics, replicability, reproducibility, soft robotics

## Abstract

Reproducibility is a particular challenge for soft robotics, yet remains a core part of its development and maturation as a field. This perspective dives into reproducibility: what it is, what it means, and how it can be applied to soft robotics. We first discuss reproducibility and delineate why it is a critical consideration for the field. Following this, our core contributions are in defining three moonshot goals that collectively chart a path towards a reproducible future for soft robotics. First, methods for testing and sharing data are discussed. Second, we show how testing procedures from other scientific disciplines can provide broad coverage over different types of soft robotics tests that we might want to complete. Finally, we highlight the need for methods to quantitatively compare the embodied intelligence that lies at the heart of soft robotics research. If successful, these steps would put the field in an excellent position to develop into the future.

## 1 Introduction

The concept of a moonshot serves as a somewhat visceral reminder of the frontier innovation that appeals to the inner scientist. Conjuring images of rapid acceleration, leaving the atmosphere strapped to an insulated calculator, venturing into the unknown … this is the type of research that many want to be doing. Moonshots should be somehow feasible, and yet also daring, fearless, and risky.

It may surprise some, then, to see the sometimes banal-sounding topic of reproducibility appearing in a special topic on Moonshots for Soft Robotics. Bare with me, and I’ll show you why reproducibility is a moonshot! In fact, I’ll show that if this daring, risky moonshot is successful, reproducibility will *become* banal for researchers in the field of soft robotics. ‘Reproducibility by default, reproducibility for all’ is the goal.

First, let’s go over the essentials. Reproducibility is critically important for soft robotics. The IEEE Soft Robotics Technical Committee has only two working groups. One is diversity and inclusion^1^, and the other is reproducibility^2^. Combined, they represent perhaps the two most valuable aspects of our wonderful field, and both are worthy of special attention. Before we continue, please note that although I am a co-chair of the Reproducibility Working Group, this article is personal opinion only. Also note that I’m solely discussing reproducibility, as opposed to benchmarking or standardisation. These are parts of the same ecosystem, however benchmarks (e.g.,Hardman et al., 2025; Greenland et al., 2025; Bhatia et al., 2021) are limited if not reproducible, and standards much more laborious and questionably necessary (as in we do not yet know which parts of soft robotics would benefit from official standardisation; although robotic manipulation has recently successfully produced a set of standards, so it is not completely impossible). However, the first step is reproducibility.

And what is that, exactly? It is somewhat ironic that the term is not commonly defined across scientific disciplines, and sometimes are not even consistent within a discipline. It is also closely tied to (but occasionally distinct from) replicability (Barba, 2018), which is again variously defined. The interested reader is referred to (Plesser, 2018) for a more expansive discussion.

For the sake of this article, we will take a common definition of reproducibility as ‘the extent to which consistent results are obtained when an experiment is repeated’, which allows others to re-run and verify results.

This definition is chosen for a few different reasons. Firstly, it is the most popular in contemporary literature and used across myriad disciplines. It is also not tied to any specific scientific discipline, meaning it should be broadly acceptable by the various disciplines that contribute to the macro-field of soft robotics.

Where a distinction between reproducibility and replicability is made in the literature, the difference is typically that reproducibility is focused on the repeating the findings using the original equipment, and replicability relates to other institutions being able to repeat the experiment. In our case, this distinction serves no purpose, and combined with a popular definition also drawing no distinction, we will define replicability as being identical to reproducibility. The decision to make reproducibility and replicability equivalent is not only a scientifically valid comparison, it also makes the following discussion easier to follow without falling into excess complexity.

Being able to repeat an experiment is a combination of experimental design, reporting, and the ability to control independent variables.

Experimental design means that the experiment is fundamentally set up to be reproducible. It uses standard equipment in standard ways. It is sufficiently observable and measurable. Measured variables can be measured with sufficient precision. The process under investigation is not sensitive to initial conditions that cannot be controlled.

Reporting means that an experiment is reported with transparency, detail, and clarity, such that that the process can be fully reproduced. There are no missing details, no poorly-defined or confusing descriptions - everything is laid out step by step. Data is provided in sufficient quantity, and analysis techniques are clear. ASTM or other standard tests are used where possible, and where not possible any bespoke equipment or testing is fully described.

Full control of independent variables is the final piece of the puzzle. The experiment must have all independent variables fully controlled. If the experiment is more complex than the experimenter envisioned, uncontrolled variables may lead to different results, even if the protocol is properly followed.

If we have all of these things, other researchers should be able to reproduce that experiment, and get the same results. The research will be reproducible.

### 1.1 Specific issues with reproducibility in soft robotics

Soft robotics has a unique relationship with reproducibility. First, Soft Robotics is young compared to robotics as a whole. Research is exploratory, and researchers are still investigating the possibilities of the field. Techniques are constantly refined and new directions emerge rapidly. In this context, it is unsurprising that the structure and framework around reproducibility are not fully in place. It also presents an opportunity that is seldom afforded to other fields–the chance to embed reproducibility into the very fabric of our research, rather than trying to add the entire paraphernalia of reproducibility after the field has matured. Working from the ground up, and from an early point in the field’s maturation, allows us to more tightly and successfully integrate. This is ‘reproducibility by design’.

A second feature of this unique relationship is that the fundamental elements of soft robotics can be hard to reproduce. Embodied intelligence underpins the entirety of soft robotics research (Pfeifer and Bongard, 2006; Howard et al., 2019), and this implies that we study dynamic feedback loops between three entities (the body, the brain, and the environment). What’s more, these interactions may persist over varying timescales, with behaviours emerging and disappearing sporadically. Some types of behaviour (something like gripping) *can* be constrained and reproducible, but can also be incredibly difficult depending on the setup of the experiment. Behaviours such as outdoor locomotion pose sterner challenges still.

In this context, it is also difficult to say if an experiment has been faithfully reproduced or not. To what extent can two behaviours (the original and the reproduction) be said to be qualitatively or quantitatively similar? There’s a huge complexity gap between reproducing something like a tensile test (Harding et al., 1960), and reproducing an embodied system behaviour (Hauser et al., 2011). Ideally we want to compare entire systems, and we should do so via behavioural descriptors that allow us to reproduce the embodied richness our systems aim for at an appropriate level. These must function whilst respecting the inherent noise and unpredictability that comes with embodied interactions. Reproducing something like an iterative optimisation process, especially those incorporating an element of experimentation (Pinskier and Howard, 2022), presents further challenges. Optimised or not, embodied behaviours are not only hard to comparably quantify, but also require larger data regimes that can be difficult to share and maintain.

Aside from issues around reproducing a science based on embodied intelligence, we should also consider the highly diverse and interdisciplinary nature of soft robotics, which is clearly a differentiated strength compared to robotics more broadly, but also presents unique challenges when it comes to reproducibility.

Soft robotics is a melting pot of various disciplines, encompassing computer science, physics, biomechanics, materials science, evolutionary biology, and machine learning, amongst others. These fields have their associated terminology, some of which overlaps with soft robotics terminology. This leads to confusion which hampers reproducibility, as a two researchers with different research backgrounds may interpret technical terms differently, leading to ambiguity.

Typical studies in soft robotics also span multiple disciplines, each with its own reproducibility considerations. The most obvious of these is on the materials side, where deformable and hyper-elastic materials are hard to characterise, even when stable. Multi-material fabrication compounds these difficulties. Printed materials depend critically on the setup of the printer itself, and many fabrication techniques are affected by ambient temperature and humidity. To give an example, our group uses a lot of Polyjet printing, which provides great freedom in geometry and material possibilities, however polyjet material properties drastically change when exposed to UV light. Being in Brisbane, we get a lot of UV, which adds a temporal dependence to the material properties and implies the need for environmental isolation of our samples, and recording of ambient conditions. Remember when I implied that a tensile test is easy to reproduce? Even this requires some thought and procedure.

Materials can mean the materials used to construct the system, but also extend to the materials used to assess the system–the test rig. What sensors are we using to perform state estimation? What actuators? Are they expensive? Are they export controlled? Is the test rig complex to set up and use? Consideration must be given to accessibility and usability of testing equipment. This is ‘reproducibility for all’.

Software, machine learning, fabrication, experimental testing, physics simulation, and material synthesis all require unique approaches to ensuring reproducibility and may have different commonly-used conventions and platforms for sharing and reporting. Holistic reporting of a soft robotics experiment that encompasses multiple disciplines is a key consideration for the field as reproducibility may break down at any point, from material synthesis through to fabrication, model training, and testing. Reporting deficiencies typically accumulate from step to step, making the challenge of end-to-end reproducibility particularly daunting and speaks to the need for substantial effort to be dedicated by the community at large for progress to be made.

The writing is on the wall: soft robotics is at a critical juncture and nothing would stymie progress like a facsimile of the ‘reproducibility crisis’ currently gripping Behavioural Psychology (Baker, 2016). The good news is that there is a general consensus, growing acknowledgement, active discussion (through a number of workshops), and broad ideation from the community on this topic (Baines et al., 2024). The urgency, scale, and ramifications if solved, classify the operationalising of reproducibility as a ‘Moonshot’ problem.

### 1.2 Comparative Analysis

By placing soft robotics’ reproducibility in the context of a range of adjacent fields, we can highlight the unique challenges that await us, as well as finding areas of commonality where we could learn from those fields. Our chosen adjacent fields range from the closely related (robotics, machine learning), to the slightly further afield (bioengineering, wet lab science).

Robotics shares many of the complexities of soft robotics, including diverse hardware and software, a lack of completeness in experimental setups, and an onus on embodied testing which adds a layer of complexity. Robotics is further along its reproducibility journey than soft robotics and has progressed in some cases to standardisation. It also has a variety of data sets that are widely used and accepted, e.g., the YCB object dataset, however there is no universally recognised singular test set in many domains, but rather a handful of variously used options. Robotics also has the benefit of the Robot Operating System (ROS), which aids in sharing of code and provides a common framework for implementing robotics software, and in producing standard formatting for datasets and common drivers for a range of sensors and actuators. Simulation offers an easier route towards sharing of experimental setups, and software can be containerized and deployed in both simulation and reality. Robotics has a strong culture of reproducibility, with many groups sharing code and videos with the aim of uptake by others.

Like robotics, machine learning benefits heavily from containerization, and the ability to easily re-run results and share repos. Unlike soft robotics, machine learning typically does not have to deal with live experimental data, although the offline datasets it uses may be noisy or from experimental apparatus. Although highly reproducible at face value, machine learning is practically complex due to variations in data and software, varying levels of metadata and documentation, and requirements for, e.g., version tracking. Machine Learning and Robotics both have a strong culture of competitions to drive collaboration, openness, and sharing.

Bioengineering and other wet lab work is inherently complex. A sophisticated and well-developed set of tools has been developed in response to this complexity and underpins research in the field. Examples include public repositories, cataloguing of environmental conditions, techniques for environmental control, careful equipment calibration, and the availability of common workflow managers, ontologies, and protocols that provide standard ways to share and report. Many lessons can be learned from reproducibility across these disciplines (see Moonshot 2, Section 2.2).

Where does soft robotics fit into this milieu? It is arguably more complex than robotics (due to the behavioural diversity of soft systems) and machine learning (owing to a hardware component in addition to the software component), and perhaps comparable to wet lab science which, although having to deal with the variability of biological matter, typically uses simpler and more automatable testing. The key differentiator between soft robotics and these fields is that soft robotics explicitly targets embodied intelligence, which is predicated on multiple interacting components in complex environment and is therefore a significant reproducibility challenge. Given the frameworks required to conduct reproducible research in comparatively complex fields, it is suggested that we will require a similarly advanced and matured set of tools to achieve the healthiest outcomes for the field of soft robotics.

## 2 Moonshots

I’ve assembled a list of moonshots below to inspire some discussion with a view to sketching out how one or more of them might be implemented. They are ordered from shorter to longer-term. All of these require community buy-in, and community action. These can all be thought of as part of the same massively-scoped moonshot, which has the goal of making *reproducibility so ingrained into soft robotics that it becomes a boring, accepted part of any soft robotics research study*.

**FIGURE 1:**
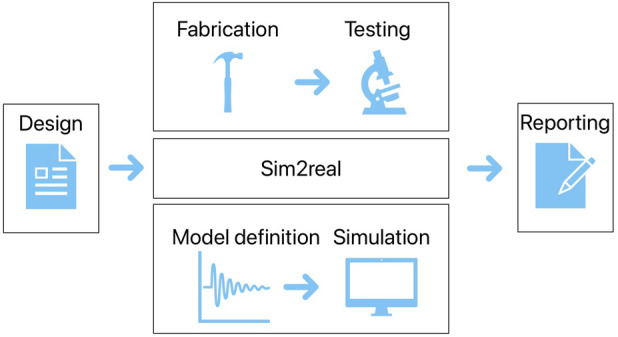
A high level diagram showing the main parts of a soft robotics experiment. Moonshots cover parts of this, in particular moonshot 2 covers the entire diagram.

To more concretely situate the scope of these moonshots, Figure 1 provides an overview of the main stages of a soft robotics experiment, from design through to reporting. In the central column, the top row corresponds to a physical experiment and the bottom row to a simulation or model-based experiment. Sim2real occupies the space between the two rows, showing how it can bridge between simulation and experimentation. Machine learning can occur anywhere in the first two columns, either as a generative design tool (first column), a modelling tool, sim2real tool, or a control generation tool (second column). Specific interventions are discussed below.

### 2.1 A kit in every lab

Testing equipment is a key influencing factor on the reproducibility of any experimental study. Unless a programmable machine such as an Instron is available, any tests, even the humble cantilever-beam, can be set up in various ways which biases results.

Protocol sharing is one part of the answer here, and protocols. io is one example of a platform to capture and share exactly how a process is enacted using various media: images, videos, notes, and so on. Going back to the ROI question, a more effective approach may be to provide test kits to labs. Numerous labs have their own setups which are consistent within a lab, what is required is some refinement (especially considering cost and materials availability) to create a cost-effective set of identical testing apparatus that can be installed at labs globally. One piece could be for manipulation, another for locomotion, another for dexterity, and so on. If combined with a set of scripts for data capture and upload, the field could move towards huge data sets with multiple contributors and unified file formats. This is ‘reproducibility by design’.

This is the most feasible of the three Moonshots, with a likely implementation pathway as follows: experimental testing kits should be designed and rolled out to a small set of research institutions; maybe 5–10 to start. Calibration tests for each kit can be used to ensure results are consistent to a known baseline. Simple scripts can be used to run experiments and collate data. Future steps may involve the initial institutions supporting the role-out of different testing kits globally. To provide maximum coverage, the experimental testing kits should be open sourced designs, able to be created on-site at many labs, or, e.g., laser cut from acrylic and shipped.

### 2.2 End-to-end testing

Tensile testing (ASTM D412) is frequently used in the field for, e.g., fitting material models. Tensile strength is a highly focused test that only provides uniaxial strain, and we rarely use other basic testing such as compressive or shear testing. Nevertheless, it forms a fundamental part of many soft robotics papers.

Tensile testing is so popular is because it is a known, trusted standard that tells us *something useful* about the material, and secondly because it can be easily done using testing machines such as the Instron, which is ubiquitous in academic and industrial settings. However the data generated is highly limited - most soft robots are not one dimensional and so would benefit more from bi- or tri-axial testing. Strain is a useful measurement, but is nowhere near enough to appropriately characterise a soft robot (more on this later).

Firstly, this moonshot seeks to expand the base tests to also include compressive and shear (e.g., ASTM D575, ASTM D945), which would tell us more about materials dynamic behaviour whilst also being well-defined and easy to replicate. It also involves looking over the fence to other fields - noting that ASTM D412 was originally designed for testing car tyres and yet has such a prominent place in soft robotics - and seeing if any other standard tests can be co-opted to benefit our field.

Taking examples from materials science, we could look at bi-axial testing using picture frame approaches (Krogh et al., 2020), diametrical compression (Procopio et al., 2003), and fracture (Dall’Asta et al., 2016). Viscoelastic testing offers yet more possibilities (Hartmann et al., 2023) to elicit fundamental material properties. Combined, they would provide a much more complete picture of material behaviour, and ideally serve as a basis to link more strongly from materials to the expectations of a given embodied behaviour if a robot were to use that material. It is important that these tests are as domain-agnostic as possible, such that more of the field can be covered, and more researchers find these characterisation methods relevant to their work. Ideally the tests could form a procedural chain which is as simple as possible, using readily-available equipment, and does not require unreasonable effort from the researcher.

Taken from our Comparative Analysis, other examples of techniques that could be co-opted by soft robotics includes the use of Github for code sharing. Machine learning in particular makes great use of Github project pages where the paper is described and linked, as well as (sometimes) interactive notepads and links to code repos. More widespread use of ROS would allow for driver sharing and common logging outputs, and increased sensorisation would allow for logging and controlling of environmental conditions (e.g., temperature, humidity) during fabrication and testing to provide a more comprehensive description of those activities to improve reproducibility.

The end goal of this moonshot is a set of tests that covers the entire ‘research lifecycle’ of a soft robotics experiment, from materials to control to machine learning to fabrication to modelling, and provides sufficient Return On Investment (ROI) that labs want to do them, either in terms of generating useful data that they can trust, or integrating other’s data and being able to trust it. We can exploit the previous effort taken by others in creating these standards, e.g., IEEE, ASTM, to increase trust and promote sharing of data, without needing to create standards ourselves. The open question is - to what extent can techniques originally developed for other fields adequately cover the needs of soft robotics? This in itself is a worthwhile exercise: it guides us to focus our efforts on any missing pieces.

Implementing this Moonshot is likely to be complex, requiring a great deal of thought and planning to ensure feasibility. It is likely to require a long time with incremental progress towards the ultimate goal. On the positive side, some of the required pieces will have already been developed and extensively tested (or even standardised) in other fields, so some of these steps could be relatively quick and proven. Implementation is likely to be in three steps: 1) identify procedures that can be usefully applied to our domain via extensive literature analysis, 2) assess those procedures within a defined assessment framework accounting for time/complexity of the intervention as well as how useful the resulting data is in general to the field, 3) keep the most useful parts and use a gap analysis to clearly define the missing parts to be created to provide a useful and usable end-to-end framework.

### 2.3 Quantitative methods to measure embodied intelligence

Soft robotics lacks a broadly applicable set of tools that help us understand, characterise, reproduce, and compare holistic and macro-scale embodied behaviours that combine dynamic morphology, control, and environmental interactions. Previous work (e.g., Hauser et al., 2011) laid some of the foundations, but it is yet to be applied wholesale across the field. It typically requires a specific set-up, and the data may not be easily available to all researchers.

This moonshot proposes a move towards behavioural characterisation, which is critically required as we are ultimately creating lifelike creatures who cannot be meaningfully quantified in any other way. Building on previous work in quantifying embodied intelligence, the moonshot would develop a set of tools for holistically characterising embodied system behaviour. Drawing together elements of deformable bodies, computational geometry, an morphological computing (emphasis on the *computing*), as well as more esoteric fields like ethology, this moonshot represents a transition from reproducible characterisation to reproducible behaviours. Core developments include quantitative frameworks (which could be task-based or environment-based) that capture key behavioural characteristics and allow us to determine if a behaviour-based experiment has been successfully reproduced or not.

Feasibility of this moonshot is the hardest to quantify, as the field of soft robotics currently has no singular agreed position on precisely what numerical measures of embodied intelligence should measure, and what they should be used for. This is why I placed this moonshot last - although robotics has strong links to embodied intelligence, the relationship is not as intrinsically intertwined as that of soft robotics and embodied intelligence, therefore there is no foundational precedent for us to co-opt. Soft robotics likely needs to be the spearhead here, leading the way to explore this currently rather nebulous concept.

Initial implementation steps would continue the ongoing research into this area, exploring potential solutions and maturing the state of the art and harnessing research into open ended evolution, quality-diversity, and novelty search (from evolutionary computing), together with pieces of behavioural science, to provide guidance and direction, providing a base set of tools that we can use to numerically quantify reasonably complex behaviours. Future iterations will improve the complexity of behaviours that can be quantified and allow for more salient measures of that behaviour to be captured; initially from complex setups but with the end goal of cheap and simple setups.

At this point we have everything we need in this critical path for reproducible soft robotics. To confirm that our moonshot has ‘landed’, we produce a singular artefact: a global soft robotics experiment characterising a behaviour, involving 50 research institutions, and comprising at least one million data evaluations. This would be an indicator that the we have succeeded. Reproducibility is now boring.

## 3 Discussion

Reproducibility is complex and multi-faceted, comprising technical, financial, behavioural and cultural elements, and conducted in a similarly complex research context. Reproducibility, alongside other important factors (Obayashi et al., 2025), must be deliberately developed if the field is to mature and grow successfully.

The path to reproducible research in soft robotics - ‘reproducibility by design, reproducibility for all’ could start with small steps. Share code, make data available, put protocols somewhere visible. Create small voluntary agreements to do something *a certain way*, and grow those networks out. Make your labs a place where metadata is a first class citizen. Share failures as well as successes. Inspire trust and confidence.

Reproducibility is a combination of experimental design, reporting, and control of independent variables. This article has shown that many of the tools and techniques are already out there and used by other fields: our first step should be to bring these in and see how well this meets our needs.

Let’s revisit the goals of this Research Topic. Firstly, we see a focus on sustainable growth, and secondly the desire to democratise the field. Hopefully I have demonstrated that reproducibility lies at the heart of both ambitions.

## Data Availability

The original contributions presented in the study are included in the article/supplementary material, further inquiries can be directed to the corresponding author.

## Footnotes

1
https://www.ieee-ras.org/soft-robotics/working-groups/diversity

2
https://www.ieee-ras.org/soft-robotics/working-groups/reproducibility
